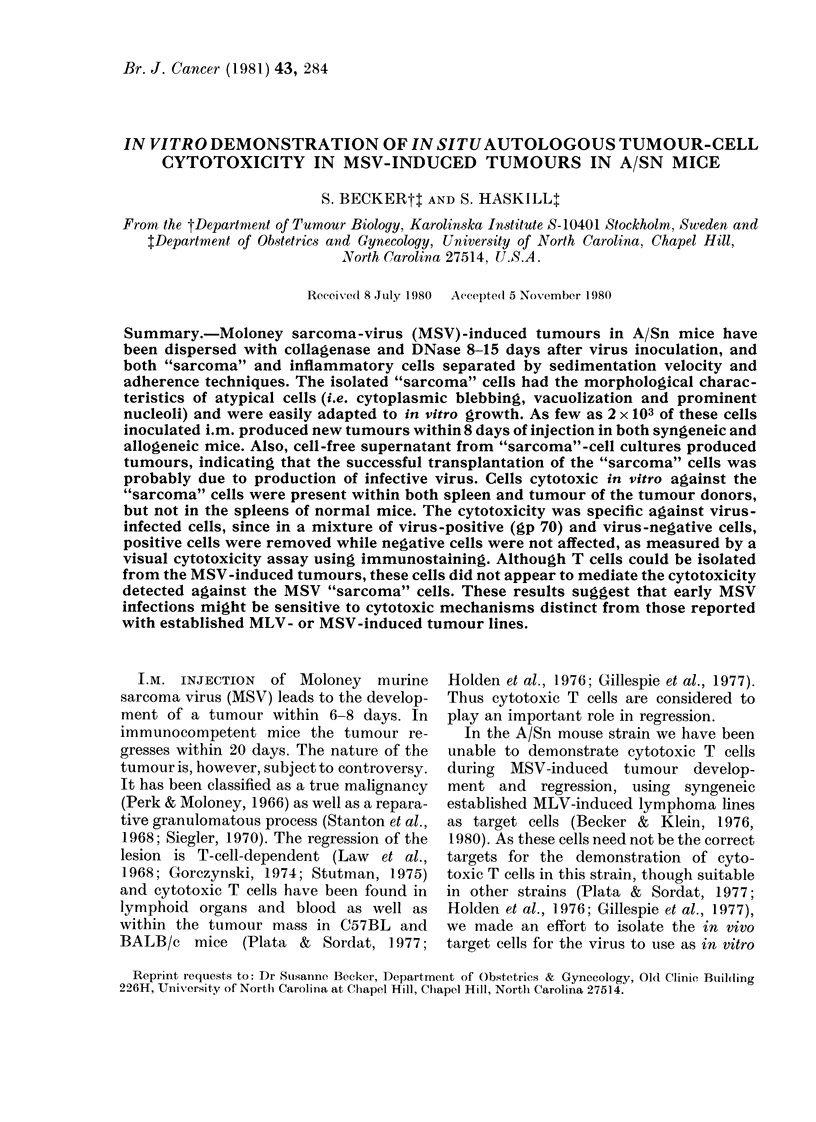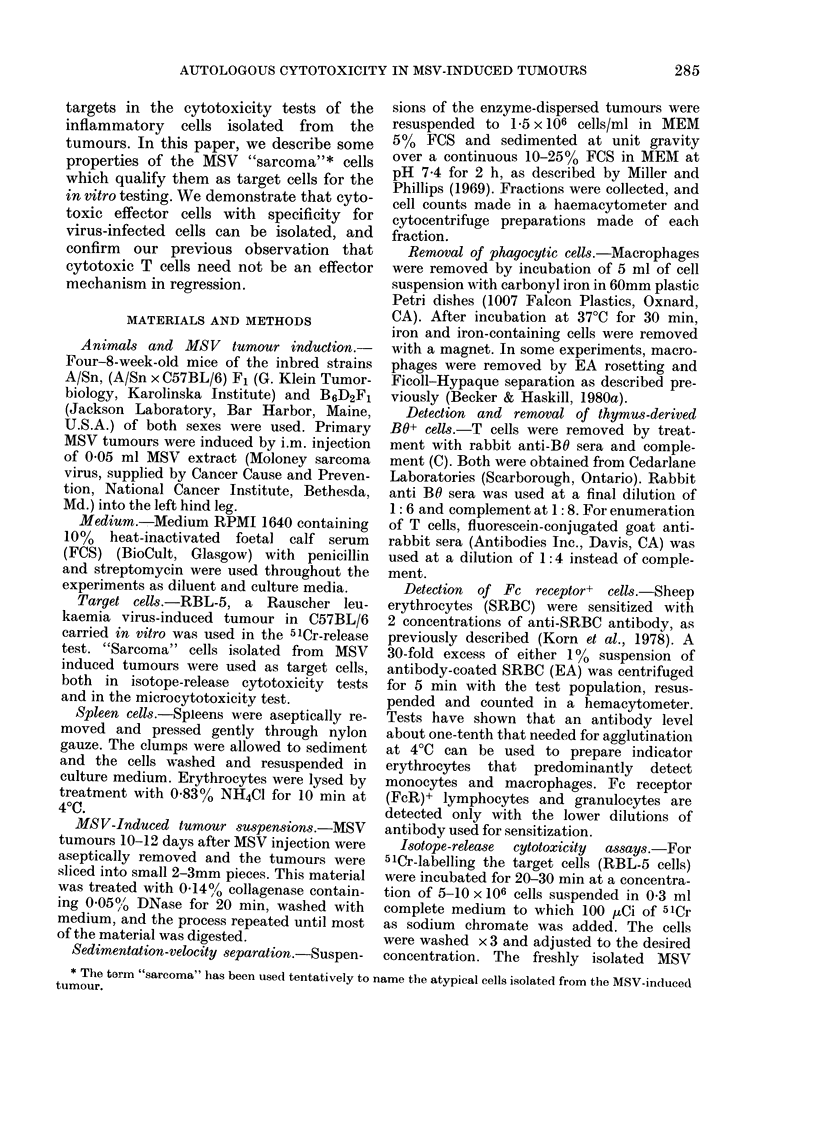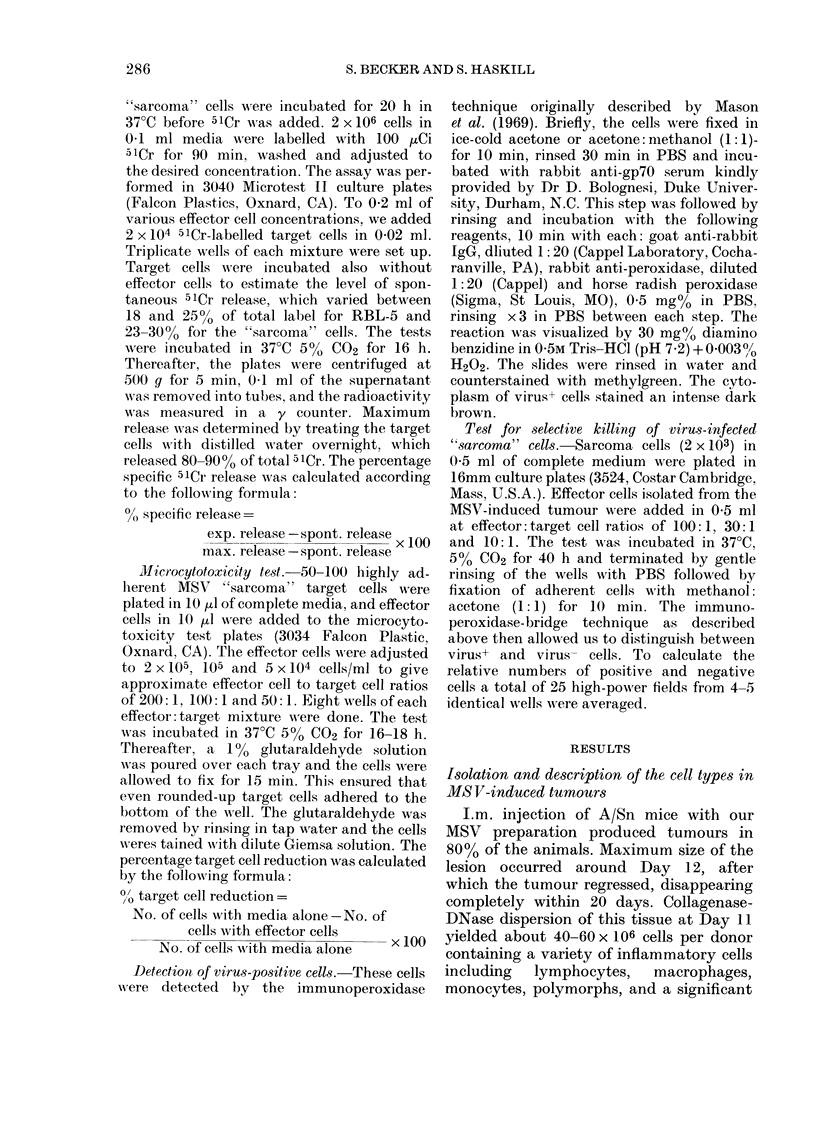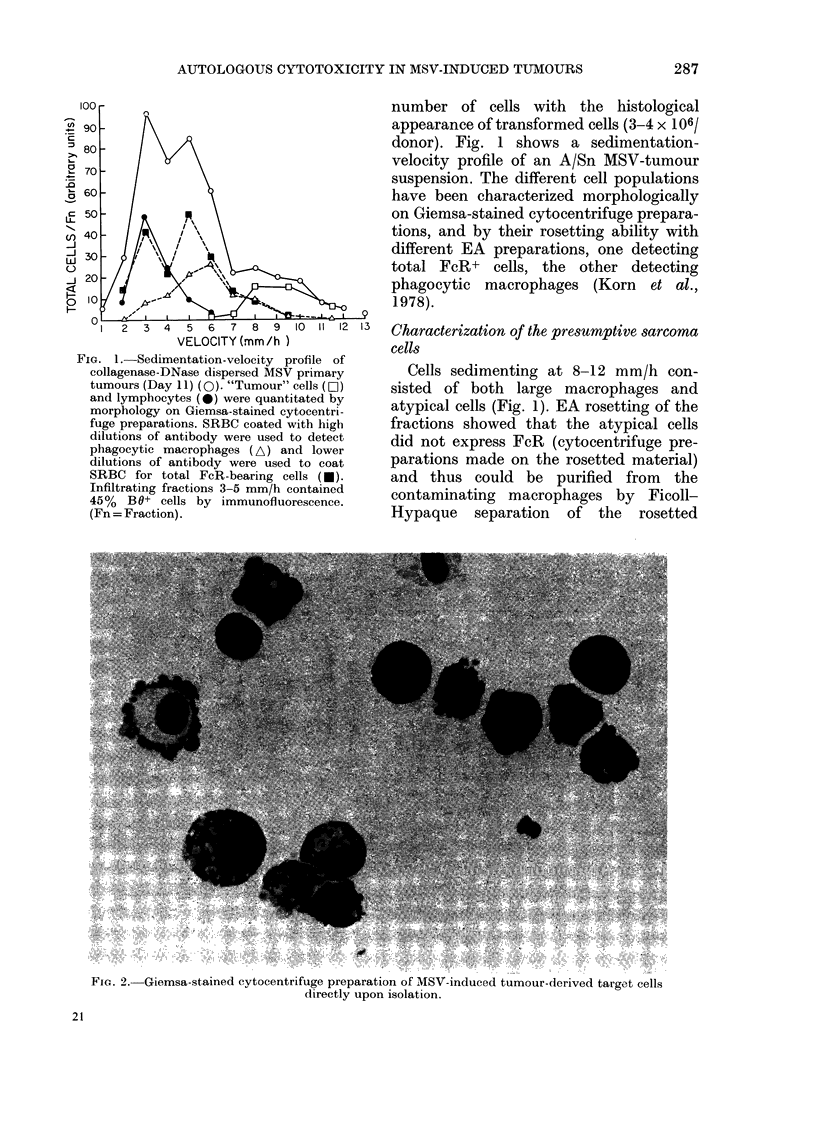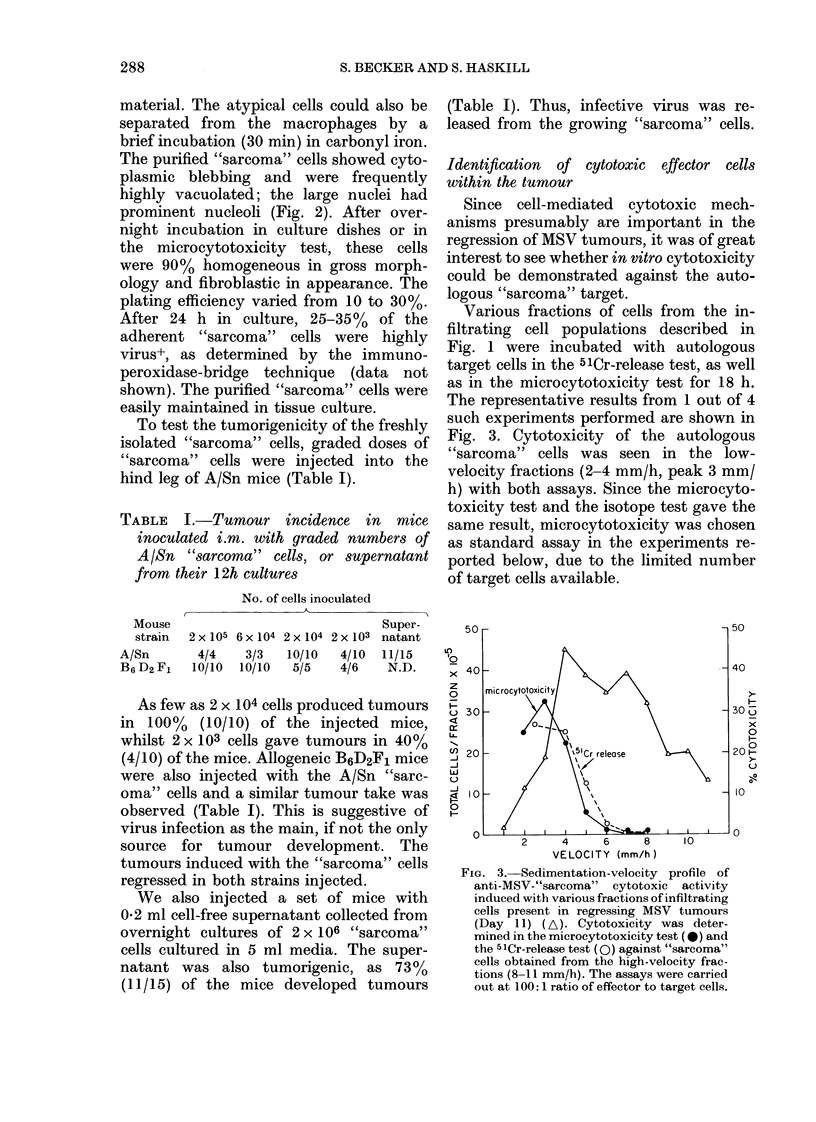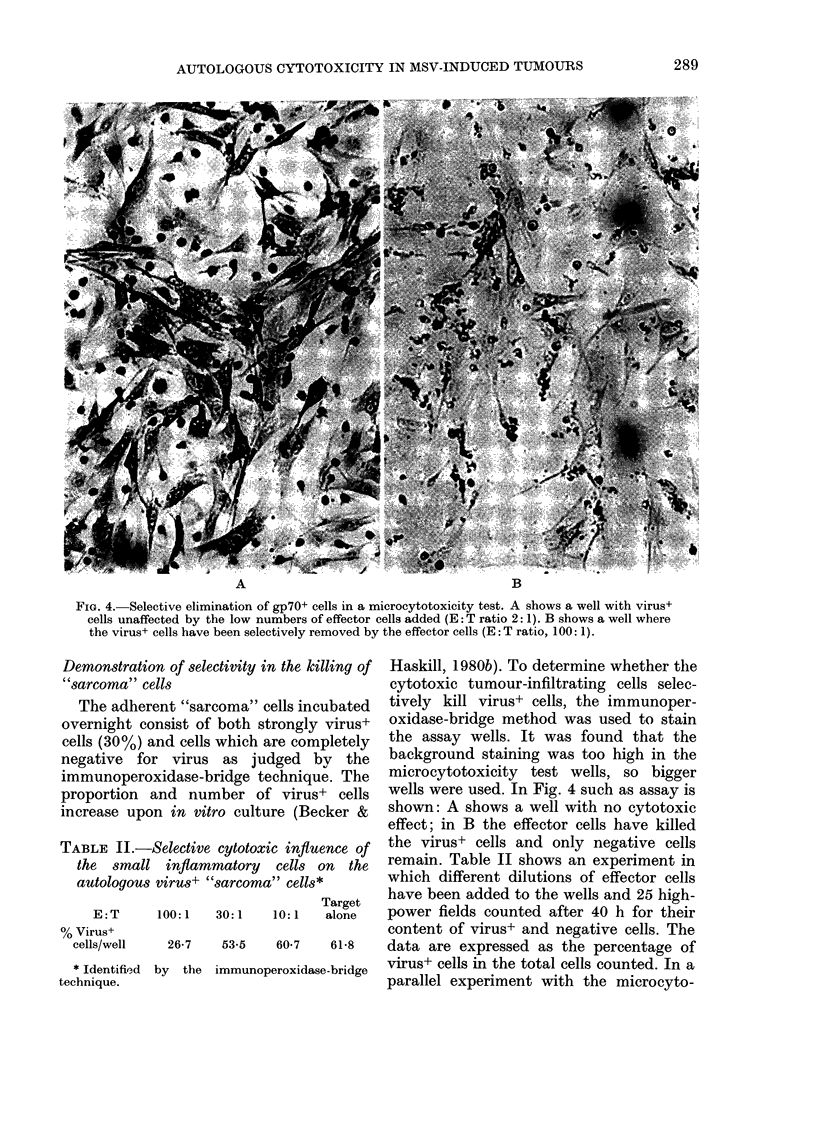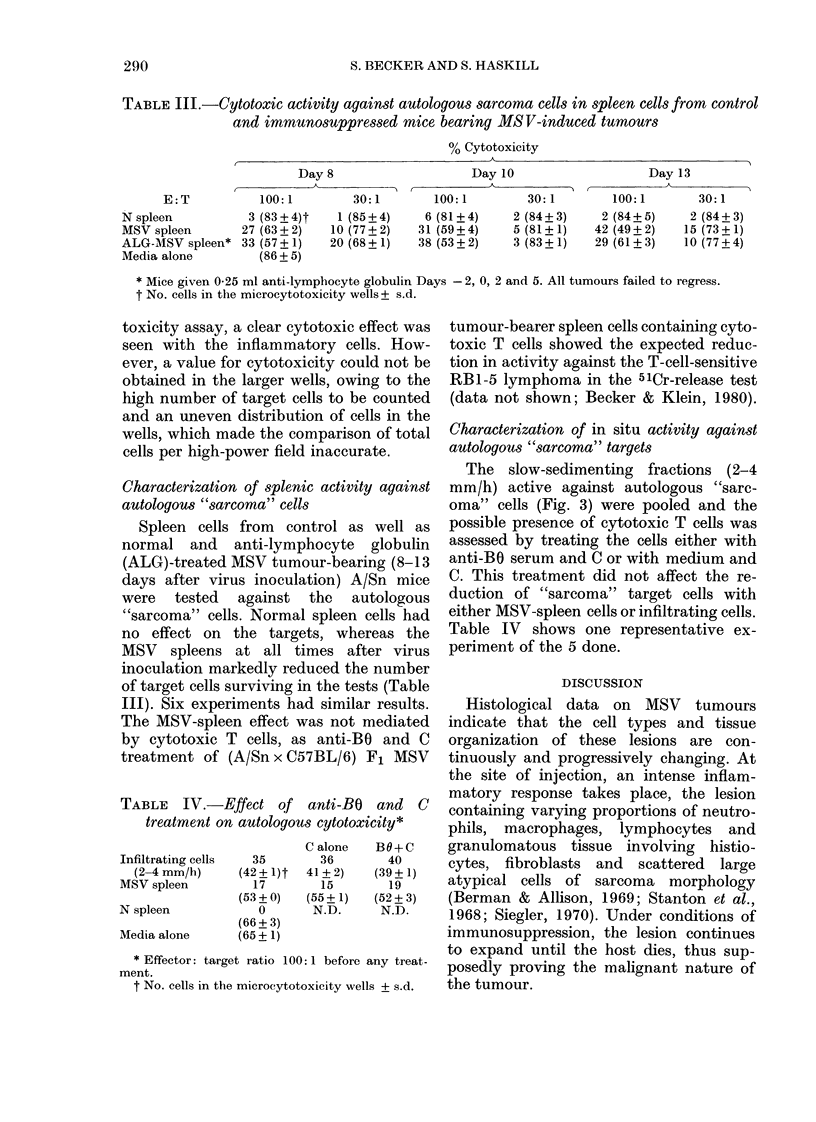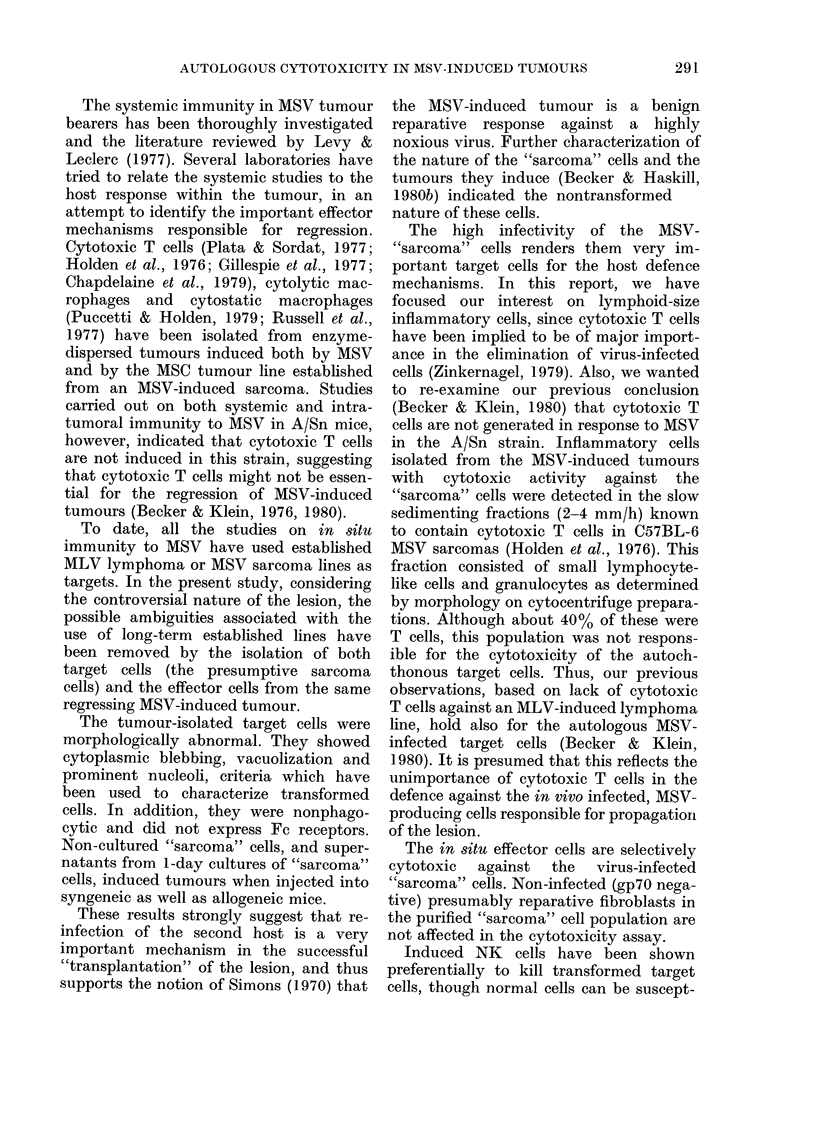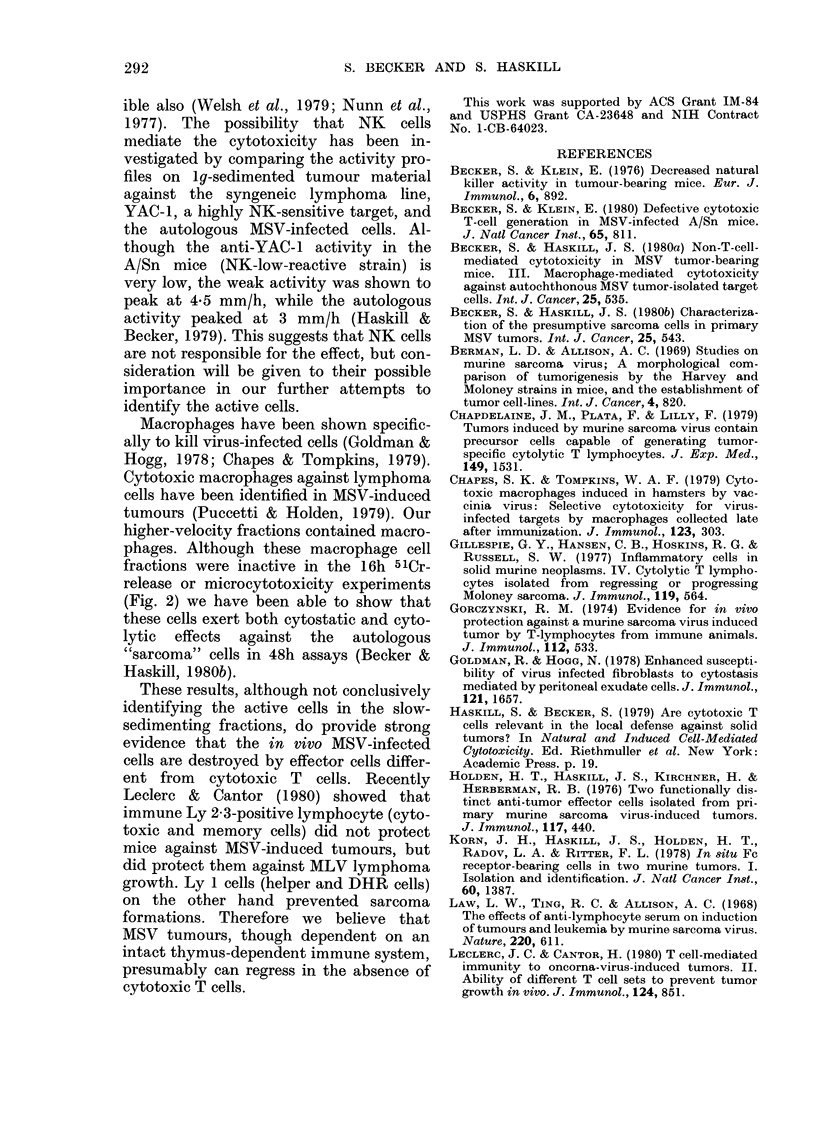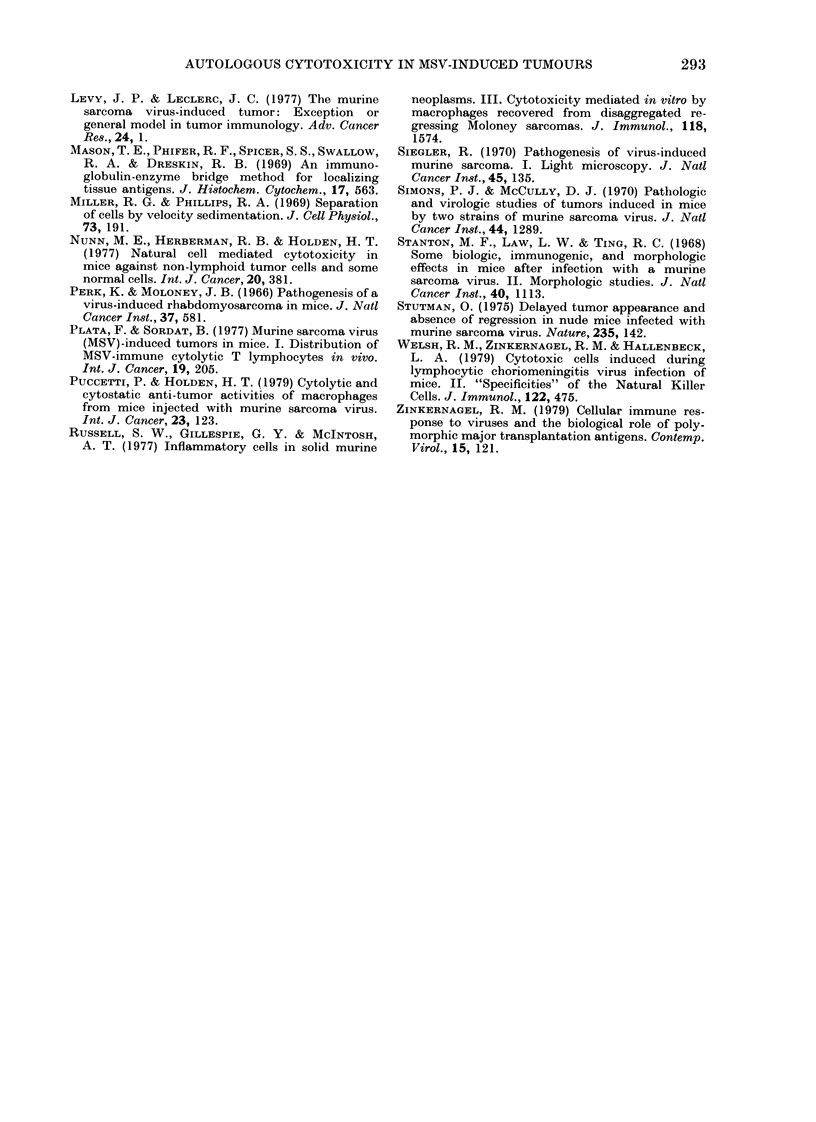# In vitro demonstration of in situ autologous tumour-cell cytotoxicity in MSV-induced tumours in A/SN mice.

**DOI:** 10.1038/bjc.1981.46

**Published:** 1981-03

**Authors:** S. Becker, S. Haskill

## Abstract

**Images:**


*Br. J. Cancer* (1981) **43**, 284

### *IN VITRO* DEMONSTRATION OF *IN SITU* AUTOLOGOUS TUMOUR-CELL CYTOTOXICITY IN MSV-INDUCED TUMOURS IN A/SN MICE

S. BECKER†‡ AND S. HASKILL‡

*From the †Department of Tumour Biology, Karolinska Institute S-10401 Stockholm, Sweden and ‡Department of Obstetrics and Gynecology, University of North Carolina, Chapel Hill, North Carolina 27514, U.S.A.*

Received 8 July 1980 Accepted 5 November 1980

**Summary.—Moloney sarcoma-virus (MSV)-induced tumours in A/Sn mice have been dispersed with collagenase and DNase 8–15 days after virus inoculation, and both "sarcoma" and inflammatory cells separated by sedimentation velocity and adherence techniques. The isolated "sarcoma" cells had the morphological characteristics of atypical cells (*i.e.* cytoplasmic blebbing, vacuolization and prominent nucleoli) and were easily adapted to *in vitro* growth. As few as $2 \times 10^3$ of these cells inoculated i.m. produced new tumours within 8 days of injection in both syngeneic and allogeneic mice. Also, cell-free supernatant from "sarcoma"-cell cultures produced tumours, indicating that the successful transplantation of the "sarcoma" cells was probably due to production of infective virus. Cells cytotoxic *in vitro* against the "sarcoma" cells were present within both spleen and tumour of the tumour donors, but not in the spleens of normal mice. The cytotoxicity was specific against virus-infected cells, since in a mixture of virus-positive (gp 70) and virus-negative cells, positive cells were removed while negative cells were not affected, as measured by a visual cytotoxicity assay using immunostaining. Although T cells could be isolated from the MSV-induced tumours, these cells did not appear to mediate the cytotoxicity detected against the MSV "sarcoma" cells. These results suggest that early MSV infections might be sensitive to cytotoxic mechanisms distinct from those reported with established MLV- or MSV-induced tumour lines.**

I.M. INJECTION of Moloney murine sarcoma virus (MSV) leads to the development of a tumour within 6–8 days. In immunocompetent mice the tumour regresses within 20 days. The nature of the tumour is, however, subject to controversy. It has been classified as a true malignancy (Perk & Moloney, 1966) as well as a reparative granulomatous process (Stanton *et al.*, 1968; Siegler, 1970). The regression of the lesion is T-cell-dependent (Law *et al.*, 1968; Gorczynski, 1974; Stutman, 1975) and cytotoxic T cells have been found in lymphoid organs and blood as well as within the tumour mass in C57BL and BALB/c mice (Plata & Sordat, 1977; Holden *et al.*, 1976; Gillespie *et al.*, 1977). Thus cytotoxic T cells are considered to play an important role in regression.

In the A/Sn mouse strain we have been unable to demonstrate cytotoxic T cells during MSV-induced tumour development and regression, using syngeneic established MLV-induced lymphoma lines as target cells (Becker & Klein, 1976, 1980). As these cells need not be the correct targets for the demonstration of cytotoxic T cells in this strain, though suitable in other strains (Plata & Sordat, 1977; Holden *et al.*, 1976; Gillespie *et al.*, 1977), we made an effort to isolate the *in vivo* target cells for the virus to use as *in vitro*

Reprint requests to: Dr Susanne Becker, Department of Obstetrics & Gynecology, Old Clinic Building 226H, University of North Carolina at Chapel Hill, Chapel Hill, North Carolina 27514.

targets in the cytotoxicity tests of the inflammatory cells isolated from the tumours. In this paper, we describe some properties of the MSV "sarcoma"* cells which qualify them as target cells for the *in vitro* testing. We demonstrate that cytotoxic effector cells with specificity for virus-infected cells can be isolated, and confirm our previous observation that cytotoxic T cells need not be an effector mechanism in regression.

### MATERIALS AND METHODS

*Animals and MSV tumour induction.*—Four–8-week-old mice of the inbred strains A/Sn, (A/Sn × C57BL/6) $F_1$ (G. Klein Tumorbiology, Karolinska Institute) and $B_6D_2F_1$ (Jackson Laboratory, Bar Harbor, Maine, U.S.A.) of both sexes were used. Primary MSV tumours were induced by i.m. injection of 0·05 ml MSV extract (Moloney sarcoma virus, supplied by Cancer Cause and Prevention, National Cancer Institute, Bethesda, Md.) into the left hind leg.

*Medium.*—Medium RPMI 1640 containing 10% heat-inactivated foetal calf serum (FCS) (BioCult, Glasgow) with penicillin and streptomycin were used throughout the experiments as diluent and culture media.

*Target cells.*—RBL-5, a Rauscher leukaemia virus-induced tumour in C57BL/6 carried *in vitro* was used in the $^{51}$Cr-release test. "Sarcoma" cells isolated from MSV induced tumours were used as target cells, both in isotope-release cytotoxicity tests and in the microcytotoxicity test.

*Spleen cells.*—Spleens were aseptically removed and pressed gently through nylon gauze. The clumps were allowed to sediment and the cells washed and resuspended in culture medium. Erythrocytes were lysed by treatment with 0·83% $NH_4Cl$ for 10 min at 4°C.

*MSV-Induced tumour suspensions.*—MSV tumours 10–12 days after MSV injection were aseptically removed and the tumours were sliced into small 2–3mm pieces. This material was treated with 0·14% collagenase containing 0·05% DNase for 20 min, washed with medium, and the process repeated until most of the material was digested.

*Sedimentation-velocity separation.*—Suspensions of the enzyme-dispersed tumours were resuspended to $1{\cdot}5 \times 10^6$ cells/ml in MEM 5% FCS and sedimented at unit gravity over a continuous 10–25% FCS in MEM at pH 7·4 for 2 h, as described by Miller and Phillips (1969). Fractions were collected, and cell counts made in a haemacytometer and cytocentrifuge preparations made of each fraction.

*Removal of phagocytic cells.*—Macrophages were removed by incubation of 5 ml of cell suspension with carbonyl iron in 60mm plastic Petri dishes (1007 Falcon Plastics, Oxnard, CA). After incubation at 37°C for 30 min, iron and iron-containing cells were removed with a magnet. In some experiments, macrophages were removed by EA rosetting and Ficoll–Hypaque separation as described previously (Becker & Haskill, 1980*a*).

*Detection and removal of thymus-derived $B\theta^+$ cells.*—T cells were removed by treatment with rabbit anti-$B\theta$ sera and complement (C). Both were obtained from Cedarlane Laboratories (Scarborough, Ontario). Rabbit anti $B\theta$ sera was used at a final dilution of 1 : 6 and complement at 1 : 8. For enumeration of T cells, fluorescein-conjugated goat anti-rabbit sera (Antibodies Inc., Davis, CA) was used at a dilution of 1 : 4 instead of complement.

*Detection of Fc receptor$^+$ cells.*—Sheep erythrocytes (SRBC) were sensitized with 2 concentrations of anti-SRBC antibody, as previously described (Korn *et al.*, 1978). A 30-fold excess of either 1% suspension of antibody-coated SRBC (EA) was centrifuged for 5 min with the test population, resuspended and counted in a hemacytometer. Tests have shown that an antibody level about one-tenth that needed for agglutination at 4°C can be used to prepare indicator erythrocytes that predominantly detect monocytes and macrophages. Fc receptor (FcR)$^+$ lymphocytes and granulocytes are detected only with the lower dilutions of antibody used for sensitization.

*Isotope-release cytotoxicity assays.*—For $^{51}$Cr-labelling the target cells (RBL-5 cells) were incubated for 20–30 min at a concentration of $5\text{–}10 \times 10^6$ cells suspended in 0·3 ml complete medium to which 100 $\mu$Ci of $^{51}$Cr as sodium chromate was added. The cells were washed ×3 and adjusted to the desired concentration. The freshly isolated MSV

* The term "sarcoma" has been used tentatively to name the atypical cells isolated from the MSV-induced tumour.

"sarcoma" cells were incubated for 20 h in 37°C before $^{51}Cr$ was added. $2 \times 10^6$ cells in 0·1 ml media were labelled with 100 $\mu$Ci $^{51}Cr$ for 90 min, washed and adjusted to the desired concentration. The assay was performed in 3040 Microtest II culture plates (Falcon Plastics, Oxnard, CA). To 0·2 ml of various effector cell concentrations, we added $2 \times 10^4$ $^{51}Cr$-labelled target cells in 0·02 ml. Triplicate wells of each mixture were set up. Target cells were incubated also without effector cells to estimate the level of spontaneous $^{51}Cr$ release, which varied between 18 and 25% of total label for RBL-5 and 23–30% for the "sarcoma" cells. The tests were incubated in 37°C 5% $CO_2$ for 16 h. Thereafter, the plates were centrifuged at 500 *g* for 5 min, 0·1 ml of the supernatant was removed into tubes, and the radioactivity was measured in a $\gamma$ counter. Maximum release was determined by treating the target cells with distilled water overnight, which released 80–90% of total $^{51}Cr$. The percentage specific $^{51}Cr$ release was calculated according to the following formula:

$$\% \text{ specific release} = \frac{\text{exp. release} - \text{spont. release}}{\text{max. release} - \text{spont. release}} \times 100$$

*Microcytotoxicity test.*—50–100 highly adherent MSV "sarcoma" target cells were plated in 10 $\mu$l of complete media, and effector cells in 10 $\mu$l were added to the microcytotoxicity test plates (3034 Falcon Plastic, Oxnard, CA). The effector cells were adjusted to $2 \times 10^5$, $10^5$ and $5 \times 10^4$ cells/ml to give approximate effector cell to target cell ratios of 200:1, 100:1 and 50:1. Eight wells of each effector:target mixture were done. The test was incubated in 37°C 5% $CO_2$ for 16–18 h. Thereafter, a 1% glutaraldehyde solution was poured over each tray and the cells were allowed to fix for 15 min. This ensured that even rounded-up target cells adhered to the bottom of the well. The glutaraldehyde was removed by rinsing in tap water and the cells weres tained with dilute Giemsa solution. The percentage target cell reduction was calculated by the following formula:

$$\% \text{ target cell reduction} = \frac{\text{No. of cells with media alone} - \text{No. of cells with effector cells}}{\text{No. of cells with media alone}} \times 100$$

*Detection of virus-positive cells.*—These cells were detected by the immunoperoxidase technique originally described by Mason *et al.* (1969). Briefly, the cells were fixed in ice-cold acetone or acetone:methanol (1:1)-for 10 min, rinsed 30 min in PBS and incubated with rabbit anti-gp70 serum kindly provided by Dr D. Bolognesi, Duke University, Durham, N.C. This step was followed by rinsing and incubation with the following reagents, 10 min with each: goat anti-rabbit IgG, dliuted 1:20 (Cappel Laboratory, Cocharanville, PA), rabbit anti-peroxidase, diluted 1:20 (Cappel) and horse radish peroxidase (Sigma, St Louis, MO), 0·5 mg% in PBS, rinsing ×3 in PBS between each step. The reaction was visualized by 30 mg% diamino benzidine in 0·5M Tris–HCl (pH 7·2)+0·003% $H_2O_2$. The slides were rinsed in water and counterstained with methylgreen. The cytoplasm of virus$^+$ cells stained an intense dark brown.

*Test for selective killing of virus-infected "sarcoma" cells.*—Sarcoma cells ($2 \times 10^3$) in 0·5 ml of complete medium were plated in 16mm culture plates (3524, Costar Cambridge, Mass, U.S.A.). Effector cells isolated from the MSV-induced tumour were added in 0·5 ml at effector:target cell ratios of 100:1, 30:1 and 10:1. The test was incubated in 37°C, 5% $CO_2$ for 40 h and terminated by gentle rinsing of the wells with PBS followed by fixation of adherent cells with methanol: acetone (1:1) for 10 min. The immunoperoxidase-bridge technique as described above then allowed us to distinguish between virus$^+$ and virus$^-$ cells. To calculate the relative numbers of positive and negative cells a total of 25 high-power fields from 4–5 identical wells were averaged.

### RESULTS

### *Isolation and description of the cell types in MSV-induced tumours*

I.m. injection of A/Sn mice with our MSV preparation produced tumours in 80% of the animals. Maximum size of the lesion occurred around Day 12, after which the tumour regressed, disappearing completely within 20 days. Collagenase-DNase dispersion of this tissue at Day 11 yielded about $40\text{–}60 \times 10^6$ cells per donor containing a variety of inflammatory cells including lymphocytes, macrophages, monocytes, polymorphs, and a significant

FIG. 1.—Sedimentation-velocity profile of collagenase-DNase dispersed MSV primary tumours (Day 11) (○). "Tumour" cells (□) and lymphocytes (●) were quantitated by morphology on Giemsa-stained cytocentrifuge preparations. SRBC coated with high dilutions of antibody were used to detect phagocytic macrophages (△) and lower dilutions of antibody were used to coat SRBC for total FcR-bearing cells (■). Infiltrating fractions 3–5 mm/h contained 45% $B\theta^+$ cells by immunofluorescence. (Fn = Fraction).

number of cells with the histological appearance of transformed cells ($3–4 \times 10^6$/donor). Fig. 1 shows a sedimentation-velocity profile of an A/Sn MSV-tumour suspension. The different cell populations have been characterized morphologically on Giemsa-stained cytocentrifuge preparations, and by their rosetting ability with different EA preparations, one detecting total $FcR^+$ cells, the other detecting phagocytic macrophages (Korn *et al.*, 1978).

*Characterization of the presumptive sarcoma cells*

Cells sedimenting at 8–12 mm/h consisted of both large macrophages and atypical cells (Fig. 1). EA rosetting of the fractions showed that the atypical cells did not express FcR (cytocentrifuge preparations made on the rosetted material) and thus could be purified from the contaminating macrophages by Ficoll–Hypaque separation of the rosetted

FIG. 2.—Giemsa-stained cytocentrifuge preparation of MSV-induced tumour-derived target cells directly upon isolation.

material. The atypical cells could also be separated from the macrophages by a brief incubation (30 min) in carbonyl iron. The purified "sarcoma" cells showed cytoplasmic blebbing and were frequently highly vacuolated; the large nuclei had prominent nucleoli (Fig. 2). After overnight incubation in culture dishes or in the microcytotoxicity test, these cells were 90% homogeneous in gross morphology and fibroblastic in appearance. The plating efficiency varied from 10 to 30%. After 24 h in culture, 25–35% of the adherent "sarcoma" cells were highly virus+, as determined by the immunoperoxidase-bridge technique (data not shown). The purified "sarcoma" cells were easily maintained in tissue culture.

To test the tumorigenicity of the freshly isolated "sarcoma" cells, graded doses of "sarcoma" cells were injected into the hind leg of A/Sn mice (Table I).

TABLE I.—*Tumour incidence in mice inoculated i.m. with graded numbers of A/Sn "sarcoma" cells, or supernatant from their 12h cultures*

| Mouse strain | No. of cells inoculated $2\times10^5$ | $6\times10^4$ | $2\times10^4$ | $2\times10^3$ | Supernatant |
|---|---|---|---|---|---|
| A/Sn | 4/4 | 3/3 | 10/10 | 4/10 | 11/15 |
| $B_6D_2F_1$ | 10/10 | 10/10 | 5/5 | 4/6 | N.D. |

As few as $2\times10^4$ cells produced tumours in 100% (10/10) of the injected mice, whilst $2\times10^3$ cells gave tumours in 40% (4/10) of the mice. Allogeneic $B_6D_2F_1$ mice were also injected with the A/Sn "sarcoma" cells and a similar tumour take was observed (Table I). This is suggestive of virus infection as the main, if not the only source for tumour development. The tumours induced with the "sarcoma" cells regressed in both strains injected.

We also injected a set of mice with 0·2 ml cell-free supernatant collected from overnight cultures of $2\times10^6$ "sarcoma" cells cultured in 5 ml media. The supernatant was also tumorigenic, as 73% (11/15) of the mice developed tumours (Table I). Thus, infective virus was released from the growing "sarcoma" cells.

*Identification of cytotoxic effector cells within the tumour*

Since cell-mediated cytotoxic mechanisms presumably are important in the regression of MSV tumours, it was of great interest to see whether *in vitro* cytotoxicity could be demonstrated against the autologous "sarcoma" target.

Various fractions of cells from the infiltrating cell populations described in Fig. 1 were incubated with autologous target cells in the $^{51}Cr$-release test, as well as in the microcytotoxicity test for 18 h. The representative results from 1 out of 4 such experiments performed are shown in Fig. 3. Cytotoxicity of the autologous "sarcoma" cells was seen in the low-velocity fractions (2–4 mm/h, peak 3 mm/h) with both assays. Since the microcytotoxicity test and the isotope test gave the same result, microcytotoxicity was chosen as standard assay in the experiments reported below, due to the limited number of target cells available.

FIG. 3.—Sedimentation-velocity profile of anti-MSV-"sarcoma" cytotoxic activity induced with various fractions of infiltrating cells present in regressing MSV tumours (Day 11) (△). Cytotoxicity was determined in the microcytotoxicity test (●) and the $^{51}Cr$-release test (○) against "sarcoma" cells obtained from the high-velocity fractions (8–11 mm/h). The assays were carried out at 100:1 ratio of effector to target cells.

FIG. 4.—Selective elimination of gp70+ cells in a microcytotoxicity test. A shows a well with virus+ cells unaffected by the low numbers of effector cells added (E:T ratio 2:1). B shows a well where the virus+ cells have been selectively removed by the effector cells (E:T ratio, 100:1).

*Demonstration of selectivity in the killing of "sarcoma" cells*

The adherent "sarcoma" cells incubated overnight consist of both strongly virus+ cells (30%) and cells which are completely negative for virus as judged by the immunoperoxidase-bridge technique. The proportion and number of virus+ cells increase upon *in vitro* culture (Becker & Haskill, 1980*b*). To determine whether the cytotoxic tumour-infiltrating cells selectively kill virus+ cells, the immunoperoxidase-bridge method was used to stain the assay wells. It was found that the background staining was too high in the microcytotoxicity test wells, so bigger wells were used. In Fig. 4 such as assay is shown: A shows a well with no cytotoxic effect; in B the effector cells have killed the virus+ cells and only negative cells remain. Table II shows an experiment in which different dilutions of effector cells have been added to the wells and 25 high-power fields counted after 40 h for their content of virus+ and negative cells. The data are expressed as the percentage of virus+ cells in the total cells counted. In a parallel experiment with the microcyto-

TABLE II.—*Selective cytotoxic influence of the small inflammatory cells on the autologous virus+ "sarcoma" cells**

| E:T | 100:1 | 30:1 | 10:1 | Target alone |
|---|---|---|---|---|
| % Virus+ cells/well | 26·7 | 53·5 | 60·7 | 61·8 |

* Identified by the immunoperoxidase-bridge technique.

TABLE III.—*Cytotoxic activity against autologous sarcoma cells in spleen cells from control and immunosuppressed mice bearing MSV-induced tumours*

| | % Cytotoxicity | | | | | |
|---|---|---|---|---|---|---|
| | Day 8 | | Day 10 | | Day 13 | |
| E:T | 100:1 | 30:1 | 100:1 | 30:1 | 100:1 | 30:1 |
| N spleen | 3 (83 ± 4)† | 1 (85 ± 4) | 6 (81 ± 4) | 2 (84 ± 3) | 2 (84 ± 5) | 2 (84 ± 3) |
| MSV spleen | 27 (63 ± 2) | 10 (77 ± 2) | 31 (59 ± 4) | 5 (81 ± 1) | 42 (49 ± 2) | 15 (73 ± 1) |
| ALG-MSV spleen* | 33 (57 ± 1) | 20 (68 ± 1) | 38 (53 ± 2) | 3 (83 ± 1) | 29 (61 ± 3) | 10 (77 ± 4) |
| Media alone | (86 ± 5) | | | | | |

* Mice given 0·25 ml anti-lymphocyte globulin Days −2, 0, 2 and 5. All tumours failed to regress.
† No. cells in the microcytotoxicity wells ± s.d.

toxicity assay, a clear cytotoxic effect was seen with the inflammatory cells. However, a value for cytotoxicity could not be obtained in the larger wells, owing to the high number of target cells to be counted and an uneven distribution of cells in the wells, which made the comparison of total cells per high-power field inaccurate.

*Characterization of splenic activity against autologous "sarcoma" cells*

Spleen cells from control as well as normal and anti-lymphocyte globulin (ALG)-treated MSV tumour-bearing (8–13 days after virus inoculation) A/Sn mice were tested against the autologous "sarcoma" cells. Normal spleen cells had no effect on the targets, whereas the MSV spleens at all times after virus inoculation markedly reduced the number of target cells surviving in the tests (Table III). Six experiments had similar results. The MSV-spleen effect was not mediated by cytotoxic T cells, as anti-Bθ and C treatment of (A/Sn × C57BL/6) $F_1$ MSV tumour-bearer spleen cells containing cytotoxic T cells showed the expected reduction in activity against the T-cell-sensitive RB1-5 lymphoma in the $^{51}$Cr-release test (data not shown; Becker & Klein, 1980).

TABLE IV.—*Effect of anti-Bθ and C treatment on autologous cytotoxicity**

| | | C alone | Bθ+C |
|---|---|---|---|
| Infiltrating cells | 35 | 36 | 40 |
| (2–4 mm/h) | (42 ± 1)† | 41 ± 2) | (39 ± 1) |
| MSV spleen | 17 | 15 | 19 |
| | (53 ± 0) | (55 ± 1) | (52 ± 3) |
| N spleen | 0 | N.D. | N.D. |
| | (66 ± 3) | | |
| Media alone | (65 ± 1) | | |

* Effector: target ratio 100:1 before any treatment.
† No. cells in the microcytotoxicity wells ± s.d.

*Characterization of* in situ *activity against autologous "sarcoma" targets*

The slow-sedimenting fractions (2–4 mm/h) active against autologous "sarcoma" cells (Fig. 3) were pooled and the possible presence of cytotoxic T cells was assessed by treating the cells either with anti-Bθ serum and C or with medium and C. This treatment did not affect the reduction of "sarcoma" target cells with either MSV-spleen cells or infiltrating cells. Table IV shows one representative experiment of the 5 done.

### DISCUSSION

Histological data on MSV tumours indicate that the cell types and tissue organization of these lesions are continuously and progressively changing. At the site of injection, an intense inflammatory response takes place, the lesion containing varying proportions of neutrophils, macrophages, lymphocytes and granulomatous tissue involving histiocytes, fibroblasts and scattered large atypical cells of sarcoma morphology (Berman & Allison, 1969; Stanton *et al.*, 1968; Siegler, 1970). Under conditions of immunosuppression, the lesion continues to expand until the host dies, thus supposedly proving the malignant nature of the tumour.

The systemic immunity in MSV tumour bearers has been thoroughly investigated and the literature reviewed by Levy & Leclerc (1977). Several laboratories have tried to relate the systemic studies to the host response within the tumour, in an attempt to identify the important effector mechanisms responsible for regression. Cytotoxic T cells (Plata & Sordat, 1977; Holden *et al.*, 1976; Gillespie *et al.*, 1977; Chapdelaine *et al.*, 1979), cytolytic macrophages and cytostatic macrophages (Puccetti & Holden, 1979; Russell *et al.*, 1977) have been isolated from enzyme-dispersed tumours induced both by MSV and by the MSC tumour line established from an MSV-induced sarcoma. Studies carried out on both systemic and intratumoral immunity to MSV in A/Sn mice, however, indicated that cytotoxic T cells are not induced in this strain, suggesting that cytotoxic T cells might not be essential for the regression of MSV-induced tumours (Becker & Klein, 1976, 1980).

To date, all the studies on *in situ* immunity to MSV have used established MLV lymphoma or MSV sarcoma lines as targets. In the present study, considering the controversial nature of the lesion, the possible ambiguities associated with the use of long-term established lines have been removed by the isolation of both target cells (the presumptive sarcoma cells) and the effector cells from the same regressing MSV-induced tumour.

The tumour-isolated target cells were morphologically abnormal. They showed cytoplasmic blebbing, vacuolization and prominent nucleoli, criteria which have been used to characterize transformed cells. In addition, they were nonphagocytic and did not express Fc receptors. Non-cultured "sarcoma" cells, and supernatants from 1-day cultures of "sarcoma" cells, induced tumours when injected into syngeneic as well as allogeneic mice.

These results strongly suggest that re-infection of the second host is a very important mechanism in the successful "transplantation" of the lesion, and thus supports the notion of Simons (1970) that the MSV-induced tumour is a benign reparative response against a highly noxious virus. Further characterization of the nature of the "sarcoma" cells and the tumours they induce (Becker & Haskill, 1980*b*) indicated the nontransformed nature of these cells.

The high infectivity of the MSV-"sarcoma" cells renders them very important target cells for the host defence mechanisms. In this report, we have focused our interest on lymphoid-size inflammatory cells, since cytotoxic T cells have been implied to be of major importance in the elimination of virus-infected cells (Zinkernagel, 1979). Also, we wanted to re-examine our previous conclusion (Becker & Klein, 1980) that cytotoxic T cells are not generated in response to MSV in the A/Sn strain. Inflammatory cells isolated from the MSV-induced tumours with cytotoxic activity against the "sarcoma" cells were detected in the slow sedimenting fractions (2–4 mm/h) known to contain cytotoxic T cells in C57BL-6 MSV sarcomas (Holden *et al.*, 1976). This fraction consisted of small lymphocyte-like cells and granulocytes as determined by morphology on cytocentrifuge preparations. Although about 40% of these were T cells, this population was not responsible for the cytotoxicity of the autochthonous target cells. Thus, our previous observations, based on lack of cytotoxic T cells against an MLV-induced lymphoma line, hold also for the autologous MSV-infected target cells (Becker & Klein, 1980). It is presumed that this reflects the unimportance of cytotoxic T cells in the defence against the *in vivo* infected, MSV-producing cells responsible for propagation of the lesion.

The *in situ* effector cells are selectively cytotoxic against the virus-infected "sarcoma" cells. Non-infected (gp70 negative) presumably reparative fibroblasts in the purified "sarcoma" cell population are not affected in the cytotoxicity assay.

Induced NK cells have been shown preferentially to kill transformed target cells, though normal cells can be susceptible also (Welsh *et al.*, 1979; Nunn *et al.*, 1977). The possibility that NK cells mediate the cytotoxicity has been investigated by comparing the activity profiles on 1*g*-sedimented tumour material against the syngeneic lymphoma line, YAC-1, a highly NK-sensitive target, and the autologous MSV-infected cells. Although the anti-YAC-1 activity in the A/Sn mice (NK-low-reactive strain) is very low, the weak activity was shown to peak at 4·5 mm/h, while the autologous activity peaked at 3 mm/h (Haskill & Becker, 1979). This suggests that NK cells are not responsible for the effect, but consideration will be given to their possible importance in our further attempts to identify the active cells.

Macrophages have been shown specifically to kill virus-infected cells (Goldman & Hogg, 1978; Chapes & Tompkins, 1979). Cytotoxic macrophages against lymphoma cells have been identified in MSV-induced tumours (Puccetti & Holden, 1979). Our higher-velocity fractions contained macrophages. Although these macrophage cell fractions were inactive in the 16h $^{51}$Cr-release or microcytotoxicity experiments (Fig. 2) we have been able to show that these cells exert both cytostatic and cytolytic effects against the autologous "sarcoma" cells in 48h assays (Becker & Haskill, 1980*b*).

These results, although not conclusively identifying the active cells in the slow-sedimenting fractions, do provide strong evidence that the *in vivo* MSV-infected cells are destroyed by effector cells different from cytotoxic T cells. Recently Leclerc & Cantor (1980) showed that immune Ly 2·3-positive lymphocyte (cytotoxic and memory cells) did not protect mice against MSV-induced tumours, but did protect them against MLV lymphoma growth. Ly 1 cells (helper and DHR cells) on the other hand prevented sarcoma formations. Therefore we believe that MSV tumours, though dependent on an intact thymus-dependent immune system, presumably can regress in the absence of cytotoxic T cells.

This work was supported by ACS Grant IM-84 and USPHS Grant CA-23648 and NIH Contract No. 1-CB-64023.

### REFERENCES

Becker, S. & Klein, E. (1976) Decreased natural killer activity in tumour-bearing mice. *Eur. J. Immunol.*, **6**, 892.

Becker, S. & Klein, E. (1980) Defective cytotoxic T-cell generation in MSV-infected A/Sn mice. *J. Natl Cancer Inst.*, **65**, 811.

Becker, S. & Haskill, J. S. (1980*a*) Non-T-cell-mediated cytotoxicity in MSV tumor-bearing mice. III. Macrophage-mediated cytotoxicity against autochthonous MSV tumor-isolated target cells. *Int. J. Cancer*, **25**, 535.

Becker, S. & Haskill, J. S. (1980*b*) Characterization of the presumptive sarcoma cells in primary MSV tumors. *Int. J. Cancer*, **25**, 543.

Berman, L. D. & Allison, A. C. (1969) Studies on murine sarcoma virus; A morphological comparison of tumorigenesis by the Harvey and Moloney strains in mice, and the establishment of tumor cell-lines. *Int. J. Cancer*, **4**, 820.

Chapdelaine, J. M., Plata, F. & Lilly, F. (1979) Tumors induced by murine sarcoma virus contain precursor cells capable of generating tumor-specific cytolytic T lymphocytes. *J. Exp. Med.*, **149**, 1531.

Chapes, S. K. & Tompkins, W. A. F. (1979) Cytotoxic macrophages induced in hamsters by vaccinia virus: Selective cytotoxicity for virus-infected targets by macrophages collected late after immunization. *J. Immunol.*, **123**, 303.

Gillespie, G. Y., Hansen, C. B., Hoskins, R. G. & Russell, S. W. (1977) Inflammatory cells in solid murine neoplasms. IV. Cytolytic T lymphocytes isolated from regressing or progressing Moloney sarcoma. *J. Immunol.*, **119**, 564.

Gorczynski, R. M. (1974) Evidence for *in vivo* protection against a murine sarcoma virus induced tumor by T-lymphocytes from immune animals. *J. Immunol.*, **112**, 533.

Goldman, R. & Hogg, N. (1978) Enhanced susceptibility of virus infected fibroblasts to cytostasis mediated by peritoneal exudate cells. *J. Immunol.*, **121**, 1657.

Haskill, S. & Becker, S. (1979) Are cytotoxic T cells relevant in the local defense against solid tumors? In *Natural and Induced Cell-Mediated Cytotoxicity*. Ed. Riethmuller *et al.* New York: Academic Press. p. 19.

Holden, H. T., Haskill, J. S., Kirchner, H. & Herberman, R. B. (1976) Two functionally distinct anti-tumor effector cells isolated from primary murine sarcoma virus-induced tumors. *J. Immunol.*, **117**, 440.

Korn, J. H., Haskill, J. S., Holden, H. T., Radov, L. A. & Ritter, F. L. (1978) *In situ* Fc receptor-bearing cells in two murine tumors. I. Isolation and identification. *J. Natl Cancer Inst.*, **60**, 1387.

Law, L. W., Ting, R. C. & Allison, A. C. (1968) The effects of anti-lymphocyte serum on induction of tumours and leukemia by murine sarcoma virus. *Nature*, **220**, 611.

Leclerc, J. C. & Cantor, H. (1980) T cell-mediated immunity to oncorna-virus-induced tumors. II. Ability of different T cell sets to prevent tumor growth *in vivo*. *J. Immunol.*, **124**, 851.

LEVY, J. P. & LECLERC, J. C. (1977) The murine sarcoma virus-induced tumor: Exception or general model in tumor immunology. *Adv. Cancer Res.*, **24**, 1.

MASON, T. E., PHIFER, R. F., SPICER, S. S., SWALLOW, R. A. & DRESKIN, R. B. (1969) An immunoglobulin-enzyme bridge method for localizing tissue antigens. *J. Histochem. Cytochem.*, **17**, 563.

MILLER, R. G. & PHILLIPS, R. A. (1969) Separation of cells by velocity sedimentation. *J. Cell Physiol.*, **73**, 191.

NUNN, M. E., HERBERMAN, R. B. & HOLDEN, H. T. (1977) Natural cell mediated cytotoxicity in mice against non-lymphoid tumor cells and some normal cells. *Int. J. Cancer*, **20**, 381.

PERK, K. & MOLONEY, J. B. (1966) Pathogenesis of a virus-induced rhabdomyosarcoma in mice. *J. Natl Cancer Inst.*, **37**, 581.

PLATA, F. & SORDAT, B. (1977) Murine sarcoma virus (MSV)-induced tumors in mice. I. Distribution of MSV-immune cytolytic T lymphocytes *in vivo*. *Int. J. Cancer*, **19**, 205.

PUCCETTI, P. & HOLDEN, H. T. (1979) Cytolytic and cytostatic anti-tumor activities of macrophages from mice injected with murine sarcoma virus. *Int. J. Cancer*, **23**, 123.

RUSSELL, S. W., GILLESPIE, G. Y. & MCINTOSH, A. T. (1977) Inflammatory cells in solid murine neoplasms. III. Cytotoxicity mediated *in vitro* by macrophages recovered from disaggregated regressing Moloney sarcomas. *J. Immunol.*, **118**, 1574.

SIEGLER, R. (1970) Pathogenesis of virus-induced murine sarcoma. I. Light microscopy. *J. Natl Cancer Inst.*, **45**, 135.

SIMONS, P. J. & MCCULLY, D. J. (1970) Pathologic and virologic studies of tumors induced in mice by two strains of murine sarcoma virus. *J. Natl Cancer Inst.*, **44**, 1289.

STANTON, M. F., LAW, L. W. & TING, R. C. (1968) Some biologic, immunogenic, and morphologic effects in mice after infection with a murine sarcoma virus. II. Morphologic studies. *J. Natl Cancer Inst.*, **40**, 1113.

STUTMAN, O. (1975) Delayed tumor appearance and absence of regression in nude mice infected with murine sarcoma virus. *Nature*, **235**, 142.

WELSH, R. M., ZINKERNAGEL, R. M. & HALLENBECK, L. A. (1979) Cytotoxic cells induced during lymphocytic choriomeningitis virus infection of mice. II. "Specificities" of the Natural Killer Cells. *J. Immunol.*, **122**, 475.

ZINKERNAGEL, R. M. (1979) Cellular immune response to viruses and the biological role of polymorphic major transplantation antigens. *Contemp. Virol.*, **15**, 121.